# Editorial and Miscellaneous

**Published:** 1863-04

**Authors:** 


### EDITORIAL AND MISCELLANEOUS.

*Insanity and Intemperance.*—We do not need to apologize for introducing to our pages the interesting paper of Dr. McFarland on this important subject. It was found impossible to make a satisfactory abstract of it and do the author justice. We had partially prepared a paper based upon our own knowledge of the Hopps case, as intimated in the January number of the JOURNAL. But the large experience of Dr. McFarland, combined with the advantages which his position, as Superintendent of the Illinois State Asylum for the Insane at Jacksonville, gives him, must render his views vastly more impressive and worthy of weight that anything we might advance.

At the first trial William Hopps was convicted because intemperance was proved. The facts of his insanity could not be disproved and the attempt was scarcely made. It has thus been gravely decided that intemperance is proof positive of sanity! Are intemperance and insanity incompatible? Both jury and court manifested the belief that they are.

Notwithstanding the complimentary mention of the presiding Judge in this case by Dr. McFarland, we cannot overlook his seemingly studied insult to the intelligence of the medical experts present in intimating that they were not capable of distinguishing, or neglected to distinguish, the manifestations of insanity present from those of drunkenness. In his remarks overruling the motion for a new trial, he deprecatingly insinuates that the medical experts were probably not aware of the prisoner's intemperate habits! When every one of them knew that this was the very point in issue, and consequently paid especial attention to that identical investigation!

Did space permit we could point out other important evidence of (designed?) misrepresentation of the positions assumed and statements made by the medical experts examined. One medical man, for instance, who was called for the defence against the remonstrance of some of the council and all of the experts, on the ground that he was incompetent to give an opinion in the case, blunderingly attributed the remarkably quick pulse of the prisoner to "an enlargement of the heart"! whilst every other physician expressly denied such a condition. When we mention that Drs. McFarland, Parker, Wing, N. S. Davis, Rea and others, certainly competent to judge, decided that there was no such disease, it is certainly surprising that the Court, fully cognizant as he was personally of the position of affairs, should have dignified this single blunder into a difference of opinion on the part of the experts,—and this with a life at stake. *Ab uno disce omnes.*

But William Hopps will have another trial. An insane man is not to be hanged in this age of the world, either to gratify the revenge or avarice of his family, or to put additional plumes upon a jackdaw prosecuting attorney, with saloon-political influences.

---

*Morbid Anatomy.*—That capital observer, practitioner and lecturer, Dr. Thomas K. Chambers, appositely remarks: "It is very rarely that which can be put up in a bottle, made an interesting preparation or picture of, that the patient feels, and that it is the business of your life to help him feel less. The true use of morbid anatomy is to teach physiology, not the art of medicine."

The study of morbid anatomy has contributed but a mere trifle to practical medicine, save through the light it has thrown upon physiology. Yet how many *post mortems* are performed, and sage inferences drawn from them, by those who know nothing of physiology!

The same idea is applicable to the study of chemistry, and even Materia Medica. Medicine will not progress, as the science now warrants, until physicians become throughly acquainted with physiology. It is the great want—worth infinitely more to the individual practitioner than a score of National Associations, or any whole chapter of legislative enactments for the protection of "the dignity of the profession."

A practice in these days which is not corrected by, if not indeed in large part based upon the scientific teachings of physiology, rises but little above, save in name, the baldest empiricism.

The erroneous practice often built up on the basis of mere morbid anatomy, has historically proved so destructive, that it becomes almost a question whether the world would not thus far have been better off if a *post mortem* had never been performed. Yet, with reference to its bearing on physiology, the *post mortem* can scarcely be over-rated in importance. The light shed upon medical practice has ultimated in the remediless ruin of the pitiless bloodletting, starvation, and tissue-destroying schools. And yet within the last few weeks we received the details of a case where an antediluvian practitioner in this city, as ignorant of the science as his horse of astronomy, when called upon, as he arrogantly stated, "*not to consult but to instruct*," kept the patient, a delicate lady who had just passed through a severe labor and was wholly anæmic and exhausted, upon *Rice Water and alterative doses of Calomel*, until death by inanition was the necessary result.

We denounce such a practice as without the pale of the profession, as brutal—not merely wanting accordance with the present high state of advance of the profession, but as meriting the penitentiary, and essentially damnable.

Nevertheless this ignorant beast is permitted to call himself a "regular physician," and is even admitted the privileges of consultation with some otherwise respectable men. And educated medical men are held responsible for the murderous antics of this bragging dotard—ten times a quack though a thousand antique diplomas were piled upon him!

---

*Absorption.*—Long since we read a book by Millingen termed "Curiosities of Medical Experience." It is our favored lot to add another to the catalogue.

Not a thousand miles from the "North Side," and not long since, in fact within the last month, a lady was confined—a not unusual circumstance. The Dr. was not long detained. A child was born, a placenta and belongings safely removed, a bandage placed around the abdomen *secundem artem*, and Medicus went on his way rejoicing. Not long after, he was sent for, as pains were still troublesome. An opiate was prescribed with orders to repeat p. r. n. But before long he was again sent for, and ordered, in addition to the anodyne, a poultice over the abdomen.

The visits were several times repeated, and anodynes and poultices long persevered with, but in vain. Visions of Puerperal fever, with all its horrors, cast a shadow of gloom over the household. After much long-suffering (forty-eight hours) the patient and friends became impatient, dismissed the attendant and sent for Dr. ——, who, in despite of his old bachelorhood, enjoys large repute in this and a similarly delicate class of cases. Palpation, notwithstanding the thick poultice, discovered through the abdominal walls the outline of an enlarged uterus, and *another child.* The Dr's expletive on the occasion is said to have been worthy of Abernethy in his best days, and the expression of his pleasant countenance worthy the brush of Hogarth. Suffice it to say, the child was speedily brought to the light, although, alas for the delay, not to life. Meanwhile the inquiry running around the fraternity is: Was the poultice put on to remove the uterine contents by favoring suppuration, or absorption?

---

*Cholesterine.*—J. H. Salisbury, M. D., in a series of experiments in the *Am. Jour. Med. Sci.*, April 1, 1863, found cholesterine and seroline in health as secretions, of the Salivary, Tear, Mammary and Sudorific glands; of the Testis and Ovary; of the Kidneys in Hepatic derangements; of Mucous Membranes when congested and inflamed; and in the fluid of Ascites and that of Spina Bifida. He states that the sudorific glands secrete them largely during the sweating stage of intermittent, remittent, varicella, diphtheritic conditions, typhoid fever, and *diabetes mellitus.* Seroline is also secreted in remittent. Butter, beef and hog suet contain both. Human milk previous to birth is rich in cholesterine, but no seroline was detected. After birth and during nursing the milk contains both largely. The milk of the cow is rich in both. As cholesterine has also been found in vegetables, it would seem to be a very common constituent of organic substances. It may be a peculiar product of nervous action and change, but evidently has other sources. It is a pretty subject to theorize about.

---

*Enchondroma.*—The Hahneman College of this city has, during the last winter, been edified by several clinical lectures upon what was called a case of spinal cord disease. The sugar plum gentry sat in council over it, and great was their jubilation that the unfortunate lad did not die whilst under the magic influence of doubly-dilute attenuations and triply divided infinitesimals. The potencies were used with an unsparing hand—but as the months went by without improvement, the father of the lad became impatient and ultimately called upon an intelligent physician. There is complete paralysis of one arm and nearly complete paralysis of the other. There is general wasting of the muscles of the shoulders and arms, to a strongly marked extent. With these exceptions the patient, a boy of some five or six years of age, is evidently in robust health.

Examination shows the presence of an enchondromatous or bony tumor, involving the arches of the first dorsal and last cervical vertebræ, perhaps one upon each side, or connected by outgrowth anterior to the bodies of the vertebræ. The tumor is much more prominent and larger upon the side completely paralyzed than upon the other.

The paralysis is seen to depend wholly upon the mechanical pressure of the tumor upon the brachial plexuses. The tumor was wholly undiscovered and neglected by the *ass-tute* spiritualistic practitioners.

The particular attendant in this case was recently heralded in the daily press as the author of a profound published essay on *Diphtheria.* It is suggested that he add an appendix on Paralysis.

---

*Glycerine Locally in Fevers.*—Jno. E. Ennis, M. D., of Lyons, Iowa, communicates to us his satisfactory experience in "the application of Glycerine to the lips, tongue and fauces in cases of continued fever, where the patients complain so much of these parts being dry and parched."

"It does not evaporate like water; is quite pleasant to the taste, and by applying three times a day perfect relief is obtained. It seems to dissolve the dry coating on the lips and tongue, and in one or two days they assume almost their natural condition. I have used it in the army during the last two years in numerous cases, and can speak positively of its beneficial effect."

---

*Health: Its Friends and its Foes.*—By R. D. Mussey, M. D., LL.D., late Prof. Anat. and Surgery at Dartmouth College, N. H., and of Surgery in the Medical College of Ohio

Fellow of the Am. Acad. of Arts and Sciences, &c., &c. Boston, Gould & Lincoln, 1862.

This little work contains the well known peculiar views of its venerable author upon hygienic subjects. By no means coinciding with his opinions upon certain mooted points, the long experience and profound sincerity of the author, together with his really valuable elucidation of many important topics will warrant us in commending the book to the perusal of our readers. Students and practitioners have no more imperious duty than that of acquainting themselves with all the facts bearing on the subject of hygiene. Sanitary Reform is the greatest object of thought for the present age.

---

*Lotion for the Hair.*—A. R. TERRY, M. D., of Detroit, after properly reprehending the use of the numerous oils and greases for the hair states: " An excellent cleansing lotion for the hair, when it is unhealthy and inclined to fall, is the following: ℞. Tinct. Cantharid ℥ ij; Aq. Ammon. ℥ iv; Alcohol, ℥ ij; Aq. Pur. ℥ viij; M. S. a tablespoonful of this may be added to half a pint of cold water, and the head thoroughly washed with it, either twice or thrice a week, or every day, according to the urgency of the case. The falling out of the hair will be generally checked in a week or two, and a new crop begin to appear; but the remedy should be continued for some weeks after. The mixture may be perfumed by the substitution of Cologne or Lavendar Water for the Alcohol, or by the addition of some aromatic essential oil in small quantities.

---

*Bunions.*—The same gentleman remarks that Bunions are often the result of Rheumatism or Gout. More frequently they result from the absurd custom of making boots and shoes much narrower across the toes than across the ball of the foot, an almost incurable vice of the shoemakers.

When the inflammation is of a slow or chronic character an ointment of Veratriæ will be found useful: ℞. Veratriæ, gr.

viij; Adipis ℥j; M. S. Rub this on the joint night and morning. In some cases the application of a blister, or of solid Lunar Caustic, sufficient to produce a blister, and followed by a poultice, has been found effectual in checking the inflammation.

Dilute Nitric Acid, or a strong solution of Hydrochlorate of Ammonia will often relieve mild cases. Tinct. Iodine has acquired repute for the same object. But a properly shaped boot or shoe, especially for the growing foot, is the great remedy and preventive agent.

---

*Glycerole of Tar.*—A combination of Glycerin and Tar is recommended in skin affections instead of the tar ointment of the pharmacopœia. ℞ Glycerin, ℥ vj; Tar, ℥ vj; Powdered Starch, ʒ ij. Warm the glycerin, stir in the starch, add the tar, and raise the mixture rapidly to the boiling point. Strain through a cloth, if necessary, and stir while cooling. The addition of the starch is found necessary to secure a uniform mass. It should be dark brown, perfectly smooth, and somewhat softer than the ointment.

---

*Graduates of the Medical Colleges.*—Bellevue College Hospital, 42; New York Medical College, 11; N. Y. Ophthalmic School and Hospital, 16; Med. Dep. Univ. Buffalo, 24; Med. Dep. Univ. Pennsylvania, 78; Jefferson Medical College, 82; Med. Dep. Univ. Michigan, 39; Cincinnati Coll. Med. and Surg., 25; Medical College of Ohio, 27; Starling (Columbus O.) College, 36; Long Island College Hospital, 11; *Rush Medical College*, 58.

---

*Iodurated Frictions in Pleurisy and Endocarditis.*—Prof. Delioux (*Gaz. Medicale de Paris*) strongly urges frictions of Iodine to promote removal of the plastic exudations of pleurisy, &c. He prefers to the Tinct. of Iodine a pomade which in general he finds most useful: ℞ Iodine ʒ ss; Iodide of Potassium ʒ ij; Axunge ℥ j. Mix. This combination is very active, indeed, requiring, where the skin is delicate, to be used with care, but it leads to the introduction of appreciable quantities of iodine into the system more certainly than any other mode. The skin should first be thoroughly cleansed, and the ointment rubbed in strongly for at least five minutes over a space somewhat larger than that affected. Repeated, say morning and evening. In the meantime the part should be covered with a layer of cotton wadding, over this oiled silk, and the whole secured in position by a bandage.

In twenty cases Prof. D. found this to triumph completely over the exudations. Treatment lasted from fifteen days to two months, in the last case there having been inflammation over both pleuræ.

He did not find it as useful in pericarditis, but in endocarditis the blowing sound rapidly disappeared on its use. Probably similar advantage might be gained in peritonitis, &c.

---

CAZEAUX' MDWFERY—*New Edition.*—We have been advised that this work was forwarded to our address through Wm. B. Keen & Co., booksellers, of this city. We can trace it in its transit just so far as we were enabled to trace PROF. GROSS' SURGERY, a year or two since, and then it mysteriously disappears, as did those lamented volumes. We had not even the melancholy satisfaction of losing them by loaning, as we have succeeded in distributing professional intelligence from our private library. *Non est inventus*—and we have a shrewd suspicion who (if he happens to read it,) will be be better posted in obstetrics by its possession.

We assure him it is a standard authority upon the subject of which it treats, and is richly worthy his perusal. Our loss will be his gain in this world, perhaps, but we earnestly fear not "in that which is to come."

---

VACCINE MATTER.—Fresh and reliable Vaccine Matter may be obtained by enclosing One Dollar and addressing

CHICAGO MEDICAL JOURNAL,
P. O. Drawer 5787, Chicago, Ill.

